# Ability of biofilm production and molecular analysis of *spa* and *ica* genes among clinical isolates of methicillin-resistant *Staphylococcus aureus*

**DOI:** 10.1186/s13104-020-4885-9

**Published:** 2020-01-07

**Authors:** Mitra Omidi, Farzaneh Firoozeh, Mahmood Saffari, Hossein Sedaghat, Mohammad Zibaei, Azad Khaledi

**Affiliations:** 1 Department of Microbiology and Immunology, School of Medicine, Kashan University of Medical Sciences, Kashan, Iran; 2 Dietary Supplements and Probiotic Research Center, Alborz University of Medical Sciences, Karaj, Iran; 3 Department of Parasitology and Mycology, School of Medicine, Alborz University of Medical Sciences, Karaj, Iran; 4 Evidence-Based Phytotherapy and Complementary Medicine Research Center, Alborz University of Medical Sciences, Karaj, Iran; 5 Department of Microbiology, School of Medicine, Alborz University of Medical Sciences, Karaj, 3149779453 IR Iran

**Keywords:** *Staphylococcus aureus*, MRSA, Biofilm, *ica*, *femA*, *mecA*, *spa*

## Abstract

**Objective:**

This study aimed to evaluate the phenotypic and genotypic characterization of biofilm formation and *spa* and *ica* genes among clinical isolates of methicillin-resistant *Staphylococcus aureus*.

**Result:**

This cross-sectional study was performed on 146 *Staphylococcus aureus* isolates from hospitalized patients in Isfahan Province Hospitals. MRSA isolates were confirmed using disk diffusion test with oxacillin disk and amplification of *mecA* gene by PCR assays. Ability of biofilm production was evaluated targeting the *icaA and icaD* genes. Of 146 *Staphylococcus aureus* isolates, 24 (16.4%) carried *mecA* genes and identified as MRSA strains. Strong ability of biofilm production was seen among 76.02% (111/146) *S. aureus* isolates and 87.5% (21/24) MRSA strains, respectively. Also, 75.0% (18/24) MRSA isolates carried *icaA* and *icaD* was not detected in these strains. Analysis of *spa* gene showed 70.83% (17/24) MRSA strains were *spa* positive. From which 14 and 3 strains identified with one band (150, 270, 300, 360, 400 bp) and two bands (150–300 bp), respectively. According to data obtained, the prevalence of MRSA isolates from Isfahan Province Hospitals is relatively high and a remarkable percentage of them show strong power in biofilm production. Also analysis of *spa* gene showed a fairly large diversity among MRSA strains.

## Introduction

*Staphylococcus aureus* (*S. aureus*) is one of the most common causes of hospital- and community-acquired infections [1–3]. The development of hospital–adapted MRSA clones in the world, has really been problematic [4, 5]. Resistance to methicillin in *S. aureus* strains is due to acquisition of *mecA* gene which encodes a changed penicillin-binding protein (PBP2a) [6–8].

Although *mecA* gene alone cannot be indicative of resistance to methicillin and existence of another ancillary gene especially *femA* (factors essential for methicillin-resistance) is also necessary for development of methicillin resistance [9]. The *S. aureus* protein A (SpA), as important virulence factor, is encoded by *spa* gene, which contain variable polymorphic X region [10]. The molecular characterization of X region of *spa* gene is documented as an exact method for typing of *S. aureus* strains [11].

The importance of biofilm production in pathogenesis of *S. aureus* and development of MDR strains has been documented [12, 13]. Apart from other adhesion factors, a polysaccharide intercellular adhesion (PIA) which is encoded by *ica* operon is essential for biofilm formation in staphylococci [13, 14]. The intercellular adhesion (*ica*) locus consists of *ica*ADBC operon which contains four genes encoding the main proteins required for the generation of PIA. The first two genes including *icaA* and *icaD* perform principal role in the synthesis of exopolysaccharides [14]. The product of *icaA* gene is a transmembrane protein with *N*-acetyl-glucosaminyl transferases enzymatic activity which led to synthesis of the poly-*N* acetyl glucosamine polymer [15]. It has been documented that, the product of the *icaD* gene, is essential for the most favorable enzymatic activity of the product of *icaA* gene [15, 16].

In this study methicillin-resistant *Staphylococcus aureus* strains were identified targeting *mecA* and *femA* genes and the analysis of *spa* gene among MRSA strains was done. Also the ability of biofilm formation was evaluated using genes carried by *ica* locus.

## Main text

### Materials and methods

#### Bacterial isolates

In this cross-sectional study, 146 *S. aureus* isolates were collected during June 2017 to September 2018 from hospitalized patients in Isfahan Province Hospitals. Identification of isolates as *S. aureus* strains was done by standard microbiological methods [17].

#### Phenotypic and genotypic identification of MRSA strains

For phenotypic identification of MRSA strains, susceptibility of isolates to cefoxitin (30 µg) and oxacillin (1 μg) disks (Mast, UK) were determined by disk diffusion method according to Clinical and Laboratory Standards Institute (CLSI) guidelines. The *S. aureus* strain COL and *S. aureus* ATCC 25923 were used as MRSA and methicillin-susceptible *Staphylococcus aureus* (MSSA) control strains respectively.

MRSA strains which had been identified phenotypically, confirmed by amplification of *mecA* and *femA* genes by PCR assays using specific primers [9, 19]. Genomic DNA was extracted by phenol chloroform method. PCR of *mecA* gene was done to amplify a 268 bp amplicon and reaction conditions was as follows: 6 min at 97 °C, 30 cycles of 30 s at 92 °C, 30 s at 55 °C, 45 s at 72 °C, and finally 10 min at 72 °C [20].

Also for amplification of 450 bp amplicon of *femA* gene the following PCR program was used: 94 °C for 5 min, 40 cycles of denaturation (94 °C, 30 s), annealing (55 °C, 40 s) and primer extension (72 °C, 50 s), with a final extension at 72 °C for 10 min [21].

#### Antibiotic susceptibility testing

According to the CLSI guidelines, resistance to antimicrobial agents was determined by disk diffusion method. The studied antibiotics were purchased from MAST company (Mast, UK) including: cefazolin, erythromycin, clindamycin, linezolid, trimethoprim sulfamethoxazole, vancomycin. The *S. aureus* strain ATCC 25923 was used as control. Vancomycin and oxacillin MICs of MRSA strains were determined using broth microdilution method and interpretation was done using susceptibility breakpoints according to the CLSI guidelines [18].

#### Biofilm production assays

Ability of biofilm production of all *S. aureus* isolates were determined by crystal violet staining assay [22]. As a negative control, uninoculated medium was used for determination of background OD. The average OD values were calculated for all tested strains and negative controls, and the cut-off OD value (ODc) was established. For interpretation of the results, strains were divided into the following groups: (I) OD ≤ ODc = no biofilm producer (0), (II), ODc < OD ≤ (2 × ODc) = weak biofilm producer (+ or 1), (III) (2 × ODc) < OD ≤ (4 × ODc) = moderate biofilm producer (++ or 2), (IV) (4 × ODc) < OD = strong biofilm producer (+++ or 3) [23].

#### Detection of icaA and icaD genes

The *icaA* and *icaD* genes were amplified in MRSA strains by PCR to detect 814 bp and 371 bp amplicons respectively using primers reported in reference 24. DNA amplification was carried out in a thermocycler (Eppendorf master cycler^®^, MA) with the following program: 94 °C, 5 min, followed by 50 cycles of (94 °C, 30 s, 59 °C, 30 s and 72 °C, 30 s) ending with 72 °C for 1 min [24]. After electrophoresis on 1% gel agarose the PCR products were visualized under UV transilluminator (Bio-Rad, UK).

#### Molecular analysis of spa gene

The variable polymorphic X region of the *spa* gene was amplified in MRSA strains and amplification reaction was according to previous studies [25].

#### Statistical analysis

Statistical analyses were done using SPSS software version 21 (SPSS, Inc.). Differences were considered by the Chi square (χ^2^) test and *P*-values less than 0.05 was considered statistically significant.

### Results

Of the 146 *S. aureus* isolates 64 (43.8%) and 82 (56.2%) isolates were recovered from males and females, respectively. The age of patients ranged from 4 months to 84 years with the mean age of 43.02 years. The clinical specimens included burn wound 56 (38.4%), diabetic wound infection 5 (3.4%), traumatic wounds 6 (4.1%), eye infection 49 (33.6%), urine 9 (6.2%), blood 3 (2%), brain abscess 5 (3.4%), respiratory tract infections 11 (7.5%), and other infections 2 (1.4%) (Table 1).

**Table 1:** Comparison between MRSA and MSSA isolates regarding characteristics of patients, biofilm production ability and antibiotic resistance

Factors	MRSA^a^, N (%)	MSSA^b^, N (%)	*P*-value^c^
Age			
0–25	3 (12.5)	33 (27.0)	0.33
26–50	9 (37.5)	35 (28.7)	
51–84	12 (50.0)	54 (44.3)	
Sex			
Male	13 (54.2)	51 (41.8)	0.08
Female	11 (45.8)	71 (58.2)	
Clinical specimens			
Burn wound	15 (62.4)	41 (33.6)	0.12
Diabetic wound	0 (0.0)	5 (4.1)	
Traumatic wounds	0 (0.0)	6 (4.9)	
Eye	6 (25.0)	43 (35.2)	
Urine	0 (0.0)	9 (7.4)	
Blood	1 (4.2)	2 (1.7)	
Brain abscess	1 (4.2)	4 (3.3)	
Respiratory tract	1 (4.2)	10 (8.2)	
Other	–	2 (1.6)	
Hospitals			
Kashan			0.001
Shahid Beheshti	3 (12.5)	49 (40.2)	
Isfahan			
Imam Musa Kazem (Burns hospital)	15(62.5)	41(33.6)	
Al-Zahra	2(8.3)	29(23.7)	
Amin	4(16.7)	3(2.5)	
Wards			
Burns	15(62.5)	41(33.6)	0.09
Infectious diseases	–	14(11.5)	
Emergency	7(29.2)	47(38.5)	
ICU^d^	2(8.3)	11(9.0)	
Outpatients	–	9(7.4)	
Biofilm production ability			
Strong	21 (87.5)	90 (73.7)	0.43
Moderate	1 (4.2)	5 (4.1)	
Weak	1 (4.2)	18 (14.8)	
Non-biofilm producers	1 (4.2)	9 (7.4)	
Antibiotic resistance			
MDR^e^	20 (83.3)	32 (26.2)	0.001
Non-MDR	4 (16.7)	90 (73.8)	
Total	24	122	

^a^Methicillin-resistant *Staphylococcus aureus*

^b^Methicillin-susceptible *Staphylococcus aureus*

^c^*P*< 0.05 is significant

^d^Intensive care unit

^e^Multiple-drug resistance

Among total *S. aureus* isolates, 24 (16.4%) identified as MRSA strains by disk diffusion method using cefoxitin and oxacillin disks, while the remaining 122 (83.6%) strains were MSSA. All 24 identified MRSA strains showed to carry *mecA* and *femA* genes.

The results of the antimicrobial sensitivity tests are represented in Table 2. Vancomycin resistance was not detected among the *S. aureus* isolates, although 3 (2.1%) of isolates showed resistance to linezolid. Also 52 (35.6%) of isolates identified as multidrug resistant (MDR) strains, which most of them were isolated from burn wounds and eye infections, respectively (Table 2). The results of phenotypic study of biofilm production, showed that 136 of 146 (93.1%) *S. aureus* isolates was biofilm producer, from which, 111, 6 and 19 isolates identified as strong, moderate and weak biofilm producers respectively. Also 23/24 (95.8%) MRSA strains identified as biofilm producer, from which 21 (87.5%), 1 (4.2%) and 1 (4.2%) were strong, moderate and weak biofilm producers, respectively. The results of phenotypic study of biofilm production in MSSA strains were as follow: 113 out of 122 (92.6%) MSSA isolates were biofilm producer, from which 90 (73.7%), 5 (4.1%) and 18 (14.8) identified with strong, moderate and weak biofilm production ability respectively.

**Table 2:** The antimicrobial sensitivity tests of MSSA and MRSA isolates by disk diffusion method

Antibiotic	MSSA (n = 122)			MRSA (n = 24)		
	Susceptible N (%)	Intermediate N (%)	Resistant N (%)	Susceptible N (%)	Intermediate N (%)	Resistant N (%)
Erythromycin	62 (50.8)	32 (26.2)	28 (23.0)	5 (20.8)	2 (8.4)	17 (70.8)
Clindamycin	100 (82.0)	14 (11.5)	8 (6.5)	6 (25.0)	4 (16.7)	14 (58.3)
Cefoxitin	122 (83.6)	0 (0.0)	0 (0.0)	0 (0.0)	0 (0.0)	24 (16.4)
Cefazolin	119 (97.5)	2 (1.7)	1 (0.8)	11 (45.8)	0 (0.0)	13 (54.2)
Linezolid	120 (98.4)	0 (0.0)	2 (1.6)	23 (95.8)	0 (0.0)	1 (4.2)
Trimethoprim/sulfamethoxazole	112 (91.8)	4 (3.3)	6 (4.9)	16 (66.7)	0 (0.0)	8 (33.3)

Molecular study of *icaA* and *icaD* genes among 24 MRSA strains revealed that 18 isolates carried *icaA* gene while *icaD* gene was not detected in all MRSA strains, and 6 isolates did not carry any of these two genes. Seventeen out Of 18 *icaA* positive MRSA strains, had been identified as strong biofilm producers by phenotypic method, whilst 4/6 MRSA strains that did not carry *ica* genes, were strong biofilm producer. Analysis of *spa* gene among 24 MRSA isolates showed that 17/24 MRSA strains carried *spa* genes of different types (Table 3). From which 14 *spa* positive MRSA strains identified with one band of different length including 150, 270, 300, 360, 400 bp and 3 *spa* positive MRSA strains founded to carry double bands of *spa* genes with the length of 150–300 bp. Majority of *spa* positive MRSA strains had one band with the length of 300 bp.

**Table 3:** Analysis of *spa* gene in MRSA isolates regarding to hospital and clinical specimens

*spa* band	Size (bp)	Number (%)	Sex	Clinical specimens	Hospital	Wards
No band						
	–	7 (29.1)	Male	Burn wound	IMK^a^ Burns hospital	Burns
			Male	Burn wound	IMK Burns hospital	Burns
			Male	Burn wound	IMK Burns hospital	Burns
			Female	Eye	Amin	Emergency
			Female	Eye	Amin	Emergency
			Female	Eye	Amin	Emergency
			Female	Brain abscess	Al-Zahra	ICU
One band						
	150	3 (12.5)	Male	Burn wound	IMK Burns hospital	Burns
			Male	Burn wound	IMK Burns hospital	Burns
			Female	Burn wound	IMK Burns hospital	Burns
	270	4 (16.7)	Male	Burn wound	IMK Burns hospital	Burns
			Male	Burn wound	IMK Burns hospital	Burns
			Male	Burn wound	IMK Burns hospital	Burns
			Female	Eye	Amin	Emergency
	300	4 (16.7)	Male	Blood	Shahid Beheshti	Emergency
			Male	Eye	Shahid Beheshti	Emergency
			Male	Burn wound	IMK Burns hospital	Burns
			Female	Respiratory tract	Al-Zahra	ICU
	360	2 (8.3)	Female	Eye	Shahid Beheshti	Emergency
			Female	Burn wound	IMK Burns hospital	Burns
	400	− 4.2	Female	Burn wound	IMK Burns hospital	Burns
Two band						
	150–300	3 (12.5)	Male	Burn wound	IMK Burns hospital	Burns
			Male	Burn wound	IMK Burns hospital	Burns
			Female	Burn wound	IMK Burns hospital	Burns
Total	24 (100)					

^a^Imam Musa Kazem Burns hospital

The statistical analyses showed significant correlation between MRSA isolation and MDR phenotype. Also a clear association was seen between the methicillin resistance and hospital where the clinical specimen has been isolated (*P* ˂ 0.05).

### Discussion

Our data revealed that of all *S. aureus* isolates, 16.4% identified as MRSA strains by both phenotypic and genotypic methods. The prevalence of MRSA strains varies in different geographic regions, as in a review study has been shown that the prevalence of MRSA infection ranges from 13 to 74.0% in different parts of the world [26]. In Iran, a meta-analysis study has been reported that prevalence of MRSA infections in Mazandaran, Isfahan, Markazi, Golestan and Kerman Provinces is about 20.5 percent [27, 28]. Despite there was no significant relationship between sex, clinical specimens and resistance to methicillin (*P *> 0.05), most MRSA isolates were recovered from men over 50 years old with burn wound infections. These results may be due to the fact that men are more likely to be burned because of their jobs; also antibiotic resistance is high in the bacterial strains that cause burn infections. Linezolid is a new class of antibiotics that are introduced for treatment of infections due to MRSA [29]. Linezolid-resistant *Staphylococcus aureus* (LRSA) is still very uncommon [30]. In the present study we have reported the emergence of LRSA strains in our region for the first time. The published reports of infections due to LRSA strains between the years 2001 and 2011 in different parts of world indicate a prevalence of 0.05% [31]. In studies conducted in different geographic areas of Iran, the LRSA strains have not been documented, except studies conducted in Tabriz and Mashhad [32]. Isolation of LRSA strains from important clinical specimens such as burn wound infection and diabetic foot ulcer, can be a serious threat due to the spread of these resistant strains in the hospitals. Detailed studies based on molecular typing of strains can be very beneficial in this field. The ability of *S. aureus* strains to produce biofilm due to the durability and antibiotic resistance is among the most important virulence factors [33]. Majority of our studied *S. aureus* strains had the ability to produce strong biofilm. In comparison to other studies in Iran, the ability and power of biofilm production in our strains was much higher; however, the methods used in studies also contribute to this difference [34, 35]. Since the most of our *S. aureus* strains have been isolated from hospitalized patients and clinical samples such as burn wounds, this high power of biofilm production is very important and requires more detailed studies in this regard. Although different genes are involved in biofilm production, but in contradiction to other studies, *icaD* gene was not detected in our biofilm producer MRSA strains and *icaA* gene was the gene specifically detected in our MRSA strains with the ability of strong biofilm production [36]. The interesting result in this context was that 4/6 MRSA strains that did not carry *ica* genes, were strong biofilm producers. This indicates that genes other than *ica* may have been involved in biofilm formation in these strains.

The analysis of *spa* genes showed that the length of detected genes in the present study was shorter than the length of the *spa* genes identified by Shakeri et al. in north of Iran [37]. This can be related to the differences in the pattern of distribution of *spa* types across different geographic regions. Using the sequencing method and determining the exact *spa* types of *S. aureus* strains can be helpful in proving this claim. In the current study, 6 different patterns of *spa* gene are detected. Findings of an investigation have been reported 5 different patterns of this gene among patients with staphylococcal infections [10].

### Conclusion

According to data obtained, the prevalence of MRSA strains in *S. aureus* isolates from Isfahan Province Hospitals is relatively high and a remarkable percentage of them show strong power in biofilm production. Also analysis of *spa* gene showed a fairly large diversity among MRSA strains isolated from different hospitals, although more detailed studies using sequencing and accurate typing methods such as MLST can prove this claim.

## Limitations

- The most important limitation of the present study is that the molecular study of genes involved in biofilm production has been performed only in MRSA strains and the mentioned studies have not been performed on MSSA strains.

## Data Availability

The datasets used and analyzed during the current study are available from the corresponding author on reasonable request.

## Abbreviations

MRSA: methicillin-resistant *Staphylococcus aureus*
PBP2a: penicillin-binding protein
MSCRAMMs: microbial surface components recognizing adhesive matrix molecules
SpA: *S. aureus* protein A
MDR: multi-drug resistant
PIA: polysaccharide intercellular adhesion
TSB: trypticase soy broth
CLSI: Clinical and Laboratory Standards Institute
MSSA: methicillin-susceptible *Staphylococcus aureus*
PCR: polymerase chain reaction
PBS: phosphate buffered saline
OD: optical densities
LRSA: Linezolid-resistant *Staphylococcus aureus*
MLST: multilocus sequence typing
